# Chrono-Nutrition, Chrono-Type, and the Prevalence of Type 2 Diabetes Mellitus in a Cross-Sectional Study from the EuroPean Prospective Investigation into Cancer and Nutrition (EPIC) Study

**DOI:** 10.3390/nu16162598

**Published:** 2024-08-07

**Authors:** Leila Luján-Barroso, Hernando J. Margara-Escudero, Marta Crous-Bou, José María Huerta, María-Dolores Chirlaque, Esther Molina-Montes, María José Sánchez, Marcela Guevara, Conchi Moreno-Iribas, Pilar Amiano, Olatz Mokoroa, Sonia González, Antonio Agudo, José Ramón Quirós, Paula Jakszyn

**Affiliations:** 1 Unit of Nutrition and Cancer, Cancer Epidemiology Research Program, Catalan Institute of Oncology (ICO), Bellvitge Biomedical Research Institute (IDIBELL), 08908 Barcelona, Spain; llujan@iconcologia.net (L.L.-B.); hernando.margara@gmail.com (H.J.M.-E.); marta.crous@iconcologia.net (M.C.-B.); a.agudo@iconcologia.net (A.A.); 2 Department of Public Health, Mental Health and Maternal and Child Health Nursing, Faculty of Nursing, University of Barcelona, 08907 Barcelona, Spain; 3 Department of Epidemiology, Murcia Regional Health Council-IMIB, 30120 Murcia, Spain; josem.huerta@carm.es (J.M.H.); mdolores.chirlaque@carm.es (M.-D.C.); 4 Centro de Investigación Biomédica en Red de Epidemiología y Salud Pública (CIBERESP), 28029 Madrid, Spain; memolina@ugr.es (E.M.-M.); mariajose.sanchez.easp@juntadeandalucia.es (M.J.S.); mp.guevara.eslava@navarra.es (M.G.); mc.moreno.iribas@navarra.es (C.M.-I.); 5 Sociohealth Sciences Department, Murcia University, 30100 Murcia, Spain; 6 Department of Nutrition and Food Science, University of Granada, 18071 Granada, Spain; 7 Institute of Nutrition and Food Technology (INYTA) ‘José Mataix’, Biomedical Research Centre, University of Granada, 18071 Granada, Spain; 8 Instituto de Investigación Biosanitaria ibs.GRANADA, 18012 Granada, Spain; 9 Escuela Andaluza de Salud Pública (EASP), 18011 Granada, Spain; 10 Instituto de Salud Pública y Laboral de Navarra, 31003 Pamplona, Spain; 11 Navarra Institute for Health Research (IdiSNA), 31008 Pamplona, Spain; 12 Sub Directorate for Public Health and Addictions of Gipuzkoa, Ministry of Health of the Basque Government, 20010 San Sebastian, Spain; p-amiano@euskadi.eus (P.A.); o-mokoroa@euskadi.eus (O.M.); 13 Epidemiology of Chronic and Communicable Diseases Group, BioGipuzkoa (BioDonostia) Health Research Institute, 20014 San Sebastian, Spain; 14 Department of Functional Biology, University of Oviedo, 33007 Asturias, Spain; soniagsolares@uniovi.es (S.G.); joseramon.quirosgarcia@asturias.org (J.R.Q.); 15 Blanquerna School of Health Sciences, Ramon Llull University, 08022 Barcelona, Spain

**Keywords:** chrono-nutrition, type 2 diabetes, meal timing, macronutrients, EPIC-Spain

## Abstract

**Background**: Previous studies have shown that meal timing, poor sleep quality, and chronotype may play a relevant role in the development of type 2 diabetes mellitus (T2DM). However, its relationship with macronutrients by eating occasions has not been explored deeply. **Objective**: Our aim was to estimate the association between chrono-nutrition, sleep quality, chronotype, and the prevalence of T2DM. **Methods**: This cross-sectional study included a subset of 3465 middle-aged Caucasian adults (2068 women) from the European Prospective Investigation into Cancer and Nutrition (EPIC) Spain cohort study. In the 2017–18 follow-up, we assessed chronotype, sleep quality, diet, and sociodemographic data using validated questionnaires. Further, we used blood samples to determine serum levels of glucose. We defined a case of T2DM when serum glucose concentration was ≥126 mg/dL or when participants self-reported diabetes. **Results**: A higher prevalence of T2DM was associated with poor sleep quality (OR_poor*vs*good_ = 2.90, 95% CI = 1.30, 6.28). Carbohydrate intake at breakfast was inversely associated with the prevalence of T2DM (OR = 0.75, 95% CI = 0.66, 0.85). Finally, lipid intake at breakfast was associated with a 13% higher prevalence of T2DM (OR = 1.13, 95% CI = 1.01, 1.26) for each 1 standard deviation (1-SD) increase. **Conclusions**: This study concludes that a higher content of carbohydrates at breakfast is correlated with a reduced prevalence of T2DM, while higher lipids intake at breakfast is associated with a higher prevalence of T2DM. Furthermore, poor sleep quality is a potential factor associated with an elevated prevalence of T2DM. Our results emphasize the need for prospective studies to validate and strengthen these observed associations.

## 1. Introduction

The prevalence of type 2 diabetes mellitus (T2DM) is rising worldwide; in 2019, 9% of the adult population was affected by this pathology (463 million), increasing to 10.5% in 2021, with predictions of an increase of up to 12.2% (783.2 million) by 2045 [1]. In Europe, the estimated prevalence in 2021 was 9.2% and is expected to increase to 10.4% by 2045. Multiple risk factors, including diet, central obesity, high-level serum uric acid, smoking, depression, cardiovascular disease, dyslipidemia, hypertension, aging, ethnicity, family history of diabetes, and physical inactivity, may impact glycemic control [2,3]. Understanding them is key to achieving increased quality of life and reduced mortality rates.

In recent years, chronobiology has provided new information on risk factors for T2DM, as well as obesity and metabolic syndrome [4,5,6]. Previous investigations have shown that modern society has misaligned sleep and eating patterns with biological cycles, a term known as chrono-disruption, which could promote detrimental effects on health, such as the risk of suffering chronic diseases like T2DM. Recent studies show that regularity, duration, and sleep quality seem to play an important role in glycemic control [2,7].

Emerging evidence suggests that factors affecting circadian rhythms, such as meal timing and nutrient components (chrono-nutrition), might lead to a higher risk of diabetes when the circadian clock system is desynchronized [8]. Chrono-nutrition is influenced by the individual’s chronotype, and previous studies have observed that there is poorer glycemic control when food intakes are made at night-dark hours, especially in T2DM adult patients [8,9]. Indeed, recent studies have suggested that reducing energy and carbohydrate (CHO) consumption in the evening hours and eating in synchrony with the circadian clock by shifting more energy and CHO intake to the morning hours enhances postprandial glycemia and reduces appetite and craving in individuals with metabolic syndrome and T2DM [10]. In contrast, other pieces of research suggest that a high protein meal (41% of energy from protein and 29% from carbohydrates) could have a modulating effect on postprandial glycemia in daily intakes, where it has been observed that this effect may be beneficial mainly for people who are late-night eaters since they are more likely to have altered blood sugar levels [11].

The reduction in glycemic peaks is an essential target in the treatment of T2DM, where meal timing exerts a critical influence on peripheral clocks involved in postprandial glycemia [10]. However, the timing of intake, distribution, and the type of macronutrients on glucose homeostasis remains slightly investigated. Hence, we aimed to cross-sectionally assess the association between chrono-nutrition, sleep quality, chronotype, and the prevalence of T2DM. In addition, we evaluated the interaction with relevant risk factors such as BMI, sex, and smoking status.

## 2. Materials and Methods

### 2.1. Study Design and Population

This cross-sectional study was established using a sample of the Spanish cohort of the European Prospective Investigation into Cancer and Nutrition (EPIC) study. The Spanish cohort included 41 437 Caucasian subject, ages 29–69 years who were recruited between 1992 and 1996 from five Spanish regions (Asturias, Granada, Murcia, Navarra, and Gipuzkoa). Further details on the study design and sample characteristics can be found elsewhere [12,13].

The eligible participants for this analysis were described previously [6,14] as younger than 67 years (in the case of men) and younger than 60 years (in the case of women) as of December 2015 (n = 8000). The age restriction was based on evidence showing that the effect of chronotype and social jetlag diminishes in older generations [15], while the different sex-specific cut-off points for ages allowed us to obtain a balanced sample by sex. From them, 5600 were invited to participate, and finally, a total of 4224 (75.4%) accepted to take part in this study. Furthermore, a total of 4031 (95.4%) participants underwent a physical examination by a nurse, and 3772 (89.3%) agreed to a blood sampling procedure extraction.

This study was conducted according to the guidelines of the Declaration of Helsinki, and the study protocol was approved by the Medical Ethical Committee of the Bellvitge Hospital (Barcelona). The approval code is PR073/16 and approval date is 7 April 2016. All the participants signed informed consent.

### 2.2. Data Collection

Data collection was previously described [6,14]. Briefly, between 2017 and 2018, phone interviews were performed by trained professionals to collect information on socio-demographic, reproductive, and lifestyle factors, as well as medical history, meal timing, chronotype [16], and sleeping patterns (used to define chronotype). The Horne–Ostberg’s Morningness–Eveningness Questionnaire (MEQ) and the Munich Chrono-type Questionnaire (MCTQ) were used to collect information on sleeping habits [16]. The sleeping habit information allowed us to calculate mid-sleep time corrected for sleeping on working and weekend days (MSFc), which was used to categorize participants into three chronotypes. The cut-off points of MSFc were at percentiles 10 (2:20 a.m.) and 90 (6:30 a.m.) and classified individuals’ chronotypes as follows: early type, normal type, and late type to calculate the amount of social jetlag accrued by each participant. Social jetlag was determined by estimating the difference between average sleep duration on free days and sleep duration on working days. Sleep quality information was assessed through the Pittsburgh Sleep Quality Index (PSQI) questionnaire [17], a questionnaire of 19 items that gave us information on sleep quality over the previous month. The final score ranges from 0–21 points, and poor sleep quality is considered when the punctuation is greater than five.

### 2.3. Biological Samples

Blood samples (n = 3772) were collected to determine serum levels of specific biochemical parameters (i.e., glucose, insulin, triglycerides, and cholesterol) using standard laboratory techniques. The samples were obtained in a fasting state (10–12 h since the last intake), fractioned, aliquoted, and stored at −80 °C following standard protocols [6].

### 2.4. Dietary Information

Comprehensive dietary data were collected using the validated diet history questionnaire from the Study on Nutrition and Cardiovascular Risk in Spain (DH-ENRICA^®^ [DH-E]) [18]. The DH-E questionnaire is based on a previous EPIC-validated dietary questionnaire, but it included larger numbers of items and traditional dishes and cooking methods from Spain. The DH-E considered ten occasions of eating, including those occurring immediately after waking up during the night. Meals were classified according to frequency: less than 3, 4 to 5, and more than 5 [6]. Additionally, the DH-E used food composition tables from Spain and other countries to convert foods to nutrients. Nutrient intakes were assessed for total and simple CHO, fiber, proteins (including animal and plant-based), and lipids (monounsaturated, polyunsaturated, and saturated) for the overall and specific daily intake. The percent of energy from CHO, proteins, and fats consumed at breakfast, lunch, and dinner was calculated, as well as time-dependent energy percentages relative to the daily total energy intake.

### 2.5. Outcome Variable—Type 2 Diabetes

The prevalence of T2DM was estimated based on laboratory and self-reported data. Fasting blood serum samples were used to determine levels of glucose, and self-reported diabetes was assessed during the telephone-based interview. A prevalent case of T2DM was considered when one of the following criteria was present: serum glucose concentration ≥126 mg/dL or self-reported diabetes.

### 2.6. Statistical Analyses

For continuous variables, descriptive statistics are presented as N (%) or as the median and percentiles 25 (p25) and 75 (p75). Adjusted means for dietary intake by diabetes status variables were adjusted for the center, sex, age at recruitment (years), educational level (none, primary, technical school, secondary, university, or higher), smoking history (never, former, and current), non-laboral physical activity (MET-h/week), BMI (<25, 25–<30, and ≥30), fat percentage, and hypertension (yes or no).

To estimate the odds ratio (OR) and their confidence intervals (CI), logistic regression was used to determine the associations between different exposure variables (sleep quality, chronotype, chrono-nutrition variables, and macronutrients) and the prevalence of T2DM. Macronutrients were evaluated both as categorical variables, expressed in grams/day (quintiles), and as continuous variables (1-SD increment of controls). For the assessment of the trend test, the categorical variable was used as a continuous one. All models were adjusted for potential confounders, including age at recruitment, sex, center, educational level, smoking history, non-laboral physical activity, BMI, fat percentage, and hypertension. Moreover, in the models assessing the main groups of macronutrients, adjustments were made for additional factors such as alcohol intake (yes/no) and quantity (grams/d), total energy intake (kcal/day), and sleep quality (poor/bad). We initially evaluated additional variables, including laboral and vigorous physical activity, chronotype, social jetlag, number of eating occasions, breakfast time, lunch time, and dinner time. These variables were considered based on their potential role as confounders; however, they were not included in the final models; their inclusion did not materially change the results.

Multiplicative interactions were modeled separately by sex, BMI (<25, 25–<30, and ≥30), smoking status (never, former, and current), and sleep quality (poor or good) with T2DM and evaluated using the log-likelihood ratio test. All statistical analyses were two-sided and evaluated at a significance level of 5%. Statistical analyses were performed using R 3.6.2 and SAS v 9.4.

## 3. Results

### Descriptive Statistics

This study was based on data from 3465 participants (2068 women, 60%), with a median age of 65 (61–68) years. The prevalence of T2DM was 20% (689 cases: 368 males and 321 females) when we conducted this study. The prevalence of T2DM was higher when the sleep quality was poor (28.7%) compared to good sleep quality (19.6%). It was also higher in late-type chronotype (24.2%) compared to normal-type chronotype (19%). Additionally, T2DM prevalence was higher when social jetlag was less than 30 min (21.5%) compared to more than 1:30 h (14%). Furthermore, T2DM prevalence was greater when breakfast and dinner were consumed later than 9 a.m. and later than 9 p.m., respectively, compared to earlier hours. Furthermore, prevalent T2DM cases were one year older on average, had a lower educational level, and had a higher body mass index than subjects without T2DM (Table 1).

Table 2 provides a comprehensive overview of dietary information by T2DM status, accounting for potential confounders. In terms of carbohydrates, T2DM cases showed lower adjusted means for % energy from CHO (non-cases: 41.61% vs. cases: 40.90%), total carbohydrates (non-cases: 256.62 vs. cases: 250.00), and simple carbohydrates (non-cases: 96.62 vs. cases: 90.10). Conversely, higher means were observed for the percentage of energy from proteins (non-cases:18.50% vs. cases: 18.90%) and animal protein (non-cases: 69.34 vs. cases: 71.43). When we explored meal-specific occasions, we observed that at breakfast, non-cases were characterized by a higher energy intake (non-cases: 378.42 vs. cases: 357.97) and a higher energy contribution (non-cases: 15.46% vs. cases: 14.78%). Non-cases also had a higher percentage of CHO (non-cases: 20.53% vs. cases: 19.09%), simple CHO (non-cases: 27.52% vs. cases: 24.56%), proteins (non-cases: 11.70% vs. cases: 11.20%), and plant-based proteins (non-cases: 14.25% vs. cases: 13.45%) from the total. The same differences were observed when macronutrients were evaluated as grams/breakfast. Additionally, prevalent T2DM cases showed a higher percentage of lipids at breakfast (non-cases: 28.08% vs. cases: 29.45%). At lunch, prevalent cases had a higher percentage of simple CHO from the total (non-cases: 30.20% vs. cases: 31.29%) and a higher percentage of proteins (non-cases: 20.74% vs. cases: 21.10%). However, simple CHO measured in grams/lunch was higher in no-cases. Finally, at dinner, prevalent T2DM cases showed a higher percentage of CHO (non-cases: 21.28% vs. cases: 22.24%), simple CHO (non-cases: 20.44% vs. cases: 22.14%), plant-based proteins (non-cases: 20.62 vs. cases: 21.53), and fiber (non-cases: 19.48 vs. cases: 20.86) compared to the total. When macronutrients were measured in grams/dinner, higher intakes were observed in T2DM cases for total and animal proteins and fiber.

In the multivariable logistic analysis, as shown in Table 3, a higher prevalence of T2DM was associated with poor sleep quality (OR_poor*vs*good_ = 2.90, 95% CI = 1.30, 6.28). We observed no difference in T2DM prevalence in relation to time of sleep, chronotype, social jetlag, and chrono-nutrition variables.

Association between macronutrients by eating occasions and the prevalence of T2DM (Table 4) was nearly 60% lower for participants in the highest quintile of carbohydrate intake at breakfast (OR_Q5*vs*Q1_ = 0.40, 95% CI = 0.27, 0.59; p-trend = 0.01). Increasing 1-SD from carbohydrates at breakfast was inversely associated with the prevalence of T2DM by 25% (OR = 0.75, 95% CI = 0.66, 0.85; p-trend ≤ 0.01). On the contrary, the OR for lipids at breakfast was 1.13 (95% CI = 1.01, 1.26) per 1-SD increase. No statistical significance was found for associations between macronutrients at lunch and dinner and T2DM.

## 4. Discussion

To the best of our knowledge, this is the first population-based study using a large sample that analyzes the influence of macronutrients, timing of intake, sleep quality, and the type of chronotype on T2DM, especially in a middle-aged to elderly Caucasian population in Spain.

### 4.1. Sleeping Patterns

Our findings showed a positive association between poor sleep quality and T2DM prevalence (OR = 2.90, 95% CI = 1.30, 6.28), which is in line with the results of a large cross-sectional study from China [19] and a cohort study from Korea [20], both of which reported that poor sleep quality was associated with higher odds of being diagnosed with T2DM. Further, the US NHANES linked poor sleep quality with the prevalence of clinically identified prediabetes [21]. Briefly, this positive association could be attributed to disruptions in circadian rhythm, influencing insulin sensitivity and consequently leading to an increased risk of T2DM [2]. Another possible explanation is that people who sleep poorly are generally more likely to have an unbalanced diet and eat more foods that raise blood sugar, which correlates with obesity, a risk factor for T2DM [22].

### 4.2. Chronotype

In relation to chronotype, it has been seen that chronotype changes with age, becoming more stable and earlier with increasing age [23]. In that line, we observed that 79.7% of our participants had a similar chronotype (normal type); then, we were not able to find a statistically significant association between chronotype and the prevalence of T2DM.

### 4.3. Chrono-Nutrition

Our findings regarding macronutrient intake and the prevalence of T2DM showed that a higher intake of lipids at breakfast had a positive association with the prevalence of T2DM; particularly, we observed that for each 1-SD increase in lipid intake, the prevalence of T2DM was up to 13% higher (OR = 1.13, 95% CI = 1.01, 1.26). We observed comparable results with higher intakes of protein, although the association was not statistically significant. On the contrary, T2DM prevalence was 60% lower in participants who had a higher intake of carbohydrates at breakfast (OR_Q5vsQ1_ = 0.40, 95% CI = 0.27, 0.59; p-trend = 0.01). The influence of this macronutrient (lipids and carbohydrates) distribution throughout the day on T2DM remains relatively underexplored. The China Health and Nutrition Survey (CHNS), an ongoing cohort study, observed that a higher intake of lipids at dinner compared to breakfast increased the risk of T2DM. In addition, increasing energy from carbohydrates at breakfast (5%) by reducing energy from lipids at dinner was associated with a reduced risk of T2DM [24]. A British cohort showed that eating more carbohydrates in the morning while reducing the consumption of lipids was related to a lower risk of T2DM [25] and, in general, to a lower risk of metabolic syndrome and its components [26]. A recent comprehensive literature review tried to figure out this aspect, concluding that an earlier consumption of carbohydrates might mitigate the risk of obesity, which is one of the main risk factors of T2DM. Moreover, the timing of carbohydrate intake significantly impacts glycometabolic control, with a higher proportion of carbohydrates consumed in the evening, potentially negatively influencing it [27]. These findings underscore the direct association between patterns of carbohydrate consumption and susceptibility to the development of T2DM.

### 4.4. Strengths and Limitations

The main limitation of our study was that the cross-sectional design does not allow for the determination of causality. Information on antidiabetic medication use was not available, so the prevalence of T2DM could have been underestimated. Information on genetic predisposition was not available in this study. Consequently, our findings may be affected by residual confounding due to genetic factors. Participants for this study were recruited from a convenience sample, so selection and participation bias may be present in our study. Finally, our study included participants from a Mediterranean cohort, which might hamper the extrapolation of our findings to other populations. However, we obtained information on the diet using a validated diet history, including the timing of intake and specific times of day when foods were consumed, to gather accurate dietary data.

The strengths of this study include its novelty as the first one to examine the relationship between chrono-nutrition and chronotype with T2DM prevalence in a Spanish study. Second, we assessed chronotype and sleep quality using a validated method. Third, we adjusted for potential confounding factors in the analyses, making the association reported robust.

## 5. Conclusions

Our results suggest that a higher intake of carbohydrates (CHO) and lower consumption of lipids at breakfast are associated with a lower prevalence of Type 2 Diabetes. Additionally, poor sleep quality appears to be linked to an increased prevalence of T2DM. However, it is crucial to note that these findings warrant confirmation through prospective studies with larger and more heterogeneous populations to establish a more robust and conclusive understanding of these relationships.

## Figures and Tables

**Table 1:** Characteristics of EPIC-Spain participants according to Type 2 diabetes mellitus (T2DM) prevalence.

	Type 2 Diabetes Mellitus	
	No (n = 2776; 80.1%)	Yes (n = 689; 19.8%)
Study center		
Asturias	745 (84.5)	137 (15.5)
Granada	519 (84.1)	98 (15.9)
Murcia	606 (76.5)	186 (23.5)
Navarra	355 (63.6)	203 (36.4)
Gipuzkoa	551 (89.5)	65 (10.6)
Sex		
Men	1029 (73.7)	368 (26.3)
Women	1747 (84.5)	321 (15.5)
Age (years)	65.0 (61.0–68.0)	66.0 (63.0–69.0)
Educational level		
None	457 (73.5)	165 (26.5)
Primary school	985 (78.1)	277 (21.9)
Technical studies	375 (81.2)	87 (18.8)
Secondary school	340 (82.1)	74 (17.9)
Higher education	611 (88.4)	80 (11.6)
Smoking history		
Never	1383 (80.9)	327 (19.1)
Current	345 (80.6)	83 (19.4)
Former	1045 (78.9)	279 (21.1)
BMI		
Normal	668 (88.4)	88 (11.6)
Overweight	1279 (81.8)	285 (18.2)
Obese	817 (72.8)	306 (27.2)
% Body fat	31.80 (25.70, 37.30)	31.80 (25.30, 37.20)
Non-laboral (PA, MET-h/week)	80.25 (58.60, 104.20)	73.40 (49.60, 100.60)
PA at home (MET-h/week)	42.70 (22.40, 64.75)	39.20 (19.60, 61.22
Recreational PA (MET-h/week)	34.50 (21.00, 49.89)	30.00 (19.95, 45.00)
Sleep quality		
Good	2709 (80.4)	662 (19.6)
Poor	67 (71.3)	27 (28.7)
Time of sleep (h/work-days)	7:10 (6:25, 7:55)	7:10 (6:25, 7:57)
Time of sleep (h/free-days)	7:28 (6.35, 8:10)	7:20 (6.30, 8.10)
Chronotype		
Early type	253 (78.3)	70 (21.7)
Normal type	2192 (81.0)	514 (19.0)
Late type	279 (75.8)	89 (24.2)
Social jetlag (h)		
<0:30	2002 (78.5)	549 (21.5)
0:30–1:30	562 (85.2)	98 (14.8)
>1:30	160 (86.0)	26 (14.0)
Breakfast time (h)		
≤9:00	2156 (81.0)	505 (19.0)
>9:00	619 (77.1)	184 (22.9)
Lunch time (h)		
≤14:00	1341 (78.8)	361 (21.2)
>14:00–15:00	1075 (81.2)	251 (18.9)
>15:00	201 (82.0)	44 (18.0)
Dinner time (h)		
≤21:00	906 (80.4)	221 (19.6)
21:00	747 (79.6)	191 (20.4)
>21:00	853 (79.1)	226 (29.5)
Number of eating occasions		
≤3	103 (75.7)	33 (24.3)
4–5	1228 (81.4)	281 (18.6)
>5	1445 (79.4)	375 (20.6)

PA = Physical activity; h = hour. Continuous variables are expressed as median (p25–p75), and categorical variables are expressed as n (%). Unknown values in non-cases: smoking history (0.1%), BMI (0.3%), chronotype (1.9%), social jetlag (1.5%), breakfast time (0%), lunch time (4.6%), and dinner time (7.8%). Unknown values in cases: smoking history (0%), BMI (0.3%), chronotype (2.3%), social jetlag (0.5%), breakfast time (0%), lunch time (1%), and dinner time (1.5%).

**Table 2:** Dietary intake according to Type 2 diabetes mellitus (T2DM) prevalence.

	Type 2 Diabetes Mellitus		
	No (n = 2776; 80.1%)	Yes (n = 689; 19.8%)	*p*-Value
Dietary intake at full day			
Total energy (kcal/day)	2479.1 (2465.7–2492.6)	2457.0 (2428.8–2485.1)	0.17
Ratio energy 6–12/energy 18–23	15.38 (11.48–19.28)	11.37(3.19–19.55)	0.39
% energy from CHO	41.61 (41.39–41.83)	40.90 (40.44–41.35)	0.01
% energy from proteins	18.50 (18.40–18.59)	18.90 (18.71–19.10)	<0.001
% energy from lipids	36.79 (36.59–37.00)	37.25 (36.82–37.68)	0.06
Total CHO (g/day)	256.62 (254.84–258.41)	250.00 (246.26–253.75)	<0.001
Simple CHO (g/day)	96.62 (95.53–97.71)	90.10 (87.82–92.37)	<0.001
Proteins (g/day)	114.04 (113.30–114.77)	115.55 (114.02–117.09)	0.09
Animal protein (g/day)	69.34 (68.65–70.03)	71.43 (69.99–72.87)	0.01
Plant-based protein (g/day)	44.68 (44.30–45.06)	44.14 (43.35–44.93)	0.23
Lipids (g/day)	101.76 (100.89–102.63)	102.17 (100.35–103.99)	0.69
Monounsaturated fatty acids (g/day)	46.05 (45.61–46.49)	46.00 (45.08–46.92)	0.92
Polyunsaturated fatty acid (g/day)	18.85 (18.57–19.13)	18.90 (18.31–19.48)	0.90
Saturated fatty acid (g/day)	27.46 (27.15–27.78)	27.84 (27.18–28.50)	0.32
Fiber (g/day)	37.73 (37.35–38.12)	37.44 (36.64–38.25)	0.53
Ethanol MEN			
Non-consumer Ethanol	79 (79.0)	21 (21.0)	
Ethanol (g/day)	20.44 (19.18–21.70)	18.81 (16.64–20.98)	0.21
Ethanol WOMEN			
Non-consumer Ethanol	285 (79.8)	72 (20.2)	
Ethanol (g/day)	6.90 (6.42–7.38)	6.46 (5.26–7.67)	0.52
Dietary intake at breakfast			
Total energy at breakfast (kcal/breakfast)	378.42(371.87–384.97)	357.97(344.24–371.70)	0.01
% energy contribution from breakfast	15.46 (15.20–15.72)	14.78 (14.24–15.31)	0.03
% CHO at breakfast from the total	20.53 (20.20–20.85)	19.09 (18.41–19.77)	<0.001
% simple CHO at breakfast from the total	27.52 (27.05–27.98)	24.56 (23.59–25.54)	<0.001
% proteins at breakfast from the total	11.70 (11.50–11.90)	11.20 (10.78–11.63)	0.04
% animal proteins at breakfast from the total	9.89 (9.63–10.14)	9.70 (9.16–10.24)	0.54
% plant-based proteins at breakfast from the total	14.25 (13.94–14.56)	13.45 (12.81–14.10)	0.03
% lipids at breakfast from the total	12.54 (12.21–12.86)	12.56 (11.87–13.25)	0.95
% fiber at breakfast from the total	10.65 (10.37–10.94)	9.95 (9.36–10.55)	0.04
% energy from CHO	56.61 (56.12–57.10)	53.92 (52.89–54.94)	0.00
% energy from proteins	14.67 (14.45–14.89)	14.97 (14.50–15.43)	0.26
% energy from lipids	28.08 (27.57–28.58)	29.45 (28.39–30.51)	0.02
Total CHO (g/breakfast)	52.48 (51.57–53.39)	47.61 (45.70–49.51)	<0.001
Simple CHO (g/breakfast)	26.75 (26.20–27.31)	22.44 (21.28–23.60)	<0.001
Total Proteins (g/breakfast)	13.11 (12.87–13.35)	12.68 (12.18–13.18)	0.13
Animal protein (g/breakfast)	6.78 (6.60–6.97)	6.78 (6.40–7.17)	0.99
Plant-based protein (g/breakfast)	6.32 (6.17–6.48)	5.90 (5.58–6.22)	0.02
Lipids (g/breakfast)	12.73 (12.36–13.10)	12.76 (11.99–13.53)	0.94
Monounsaturated fatty acids (g/breakfast)	5.35 (5.16–5.53)	5.28 (4.89–5.66)	0.75
Polyunsaturated fatty acid (g/breakfast)	2.22 (2.10–2.34)	2.06 (1.81–2.30)	0.25
Saturated fatty acid (g/breakfast)	3.97 (3.84–4.10)	4.27 (3.99–4.54)	0.05
Fiber (g/breakfast)	4.04 (3.91–4.17)	3.77 (3.51–4.04)	0.08
Ethanol MEN			
Non-consumer Ethanol	1018 (73.9)	359 (26.1)	
Ethanol (g/breakfast)	9.70 (0–32.06)	18.46 (0–74.97)	0.56
Ethanol WOMEN			
Non-consumer Ethanol	1741 (84.4)	321 (15.6)	
Ethanol (g/breakfast)	-	-	
Dietary intake at lunch			
Total energy at lunch (kcal/lunch)	1173.8 (1163.8–1183.7)	1153.0 (1132.1–1173.9)	0.08
% energy contribution from lunch	47.31 (46.99–47.62)	46.91 (46.25–47.57)	0.29
% CHO at lunch from the total	45.78 (45.43–46.13)	45.85 (45.12–46.59)	0.86
% simple CHO at lunch from the total	30.20 (29.77–30.62)	31.29 (30.40–32.19)	0.03
% proteins at lunch from the total	52.72 (52.39–53.05)	52.01 (51.32–52.70)	0.07
% animal proteins at lunch from the total	50.26 (49.78–50.74)	49.56 (48.56–50.56)	0.22
% plant-based proteins at lunch from the total	56.75 (56.36–57.15)	56.30 (55.47–57.14)	0.35
% fats at lunch from the total	47.32 (46.89–47.76)	46.13 (45.21–47.04)	0.02
% fiber at lunch from the total	61.13 (60.69–61.57)	60.37 (59.45–61.30)	0.15
% energy from CHO	40.18 (39.93–40.44)	39.89 (39.36–40.42)	0.33
% energy from proteins	20.74 (20.61–20.87)	21.10 (20.83–21.37)	0.02
% energy from lipids	36.32 (36.08–36.56)	36.23 (35.73–36.73)	0.75
Total CHO (g/lunch)	117.55 (116.38–118.72)	114.91 (112.46–117.36)	0.06
Simple CHO (g/lunch)	28.53 (28.07–28.99)	27.38 (26.42–28.35)	0.04
Total Proteins (g/lunch)	59.92 (59.41–60.43)	59.84 (58.77–60.90)	0.89
Animal protein (g/lunch)	34.46 (34.02–34.89)	34.86 (33.95–35.78)	0.44
Plant-based protein (g/lunch)	25.46 (25.18–25.75)	24.97 (24.37–25.57)	0.15
Total lipids (g/lunch)	47.81 (47.24–48.37)	46.77 (45.59–47.95)	0.13
Monounsaturated fatty acids (g/lunch)	23.47 (23.18–23.76)	22.96 (22.35–23.57)	0.15
Polyunsaturated fatty acid (g/lunch)	8.46 (8.31–8.62)	8.28 (7.96–8.61)	0.34
Saturated fatty acid (g/lunch)	11.50 (11.32–11.67)	11.25 (10.88–11.61)	0.23
Fiber (g/lunch)	23.06 (22.78–23.34)	22.62 (22.03–23.21)	0.19
Ethanol MEN			
Non-consumer Ethanol	202 (70.4)	85 (29.6)	
Ethanol (g/lunch)	9.10 (8.51–9.69)	9.57 (8.53–10.61)	0.45
Ethanol WOMEN			
Non-consumer Ethanol	518 (83.4)	103 (16.6)	
Ethanol (g/lunch)	3.84 (3.54–4.13)	3.66 (2.94–4.38)	0.65
Dietary intake at dinner			
Total energy at dinner (kcal/dinner)	629.78 (621.59–637.98)	646.12 (628.94–663.29)	0.10
% energy contribution from dinner	25.29 (25.00–25.58)	26.22 (25.61–26.83)	0.01
% CHO at dinner from the total	21.28 (20.99–21.56)	22.24 (21.64–22.84)	0.01
% simple CHO at dinner from the total	20.44 (20.05–20.83)	22.14 (21.33–22.96)	<0.001
% proteins at dinner from the total	27.59 (27.27–27.91)	28.29 (27.63–28.96)	0.07
% animal proteins at dinner from the total	32.19 (31.73–32.66)	32.52 (31.55–33.49)	0.56
% plant-based proteins at dinner from the total	20.62 (20.30–20.93)	21.53 (20.86–22.19)	0.02
% fats at dinner from the total	28.49 (28.06–28.91)	29.57 (28.68–30.46)	0.03
% fiber at dinner from the total	19.48 (19.14–19.82)	20.86 (20.15–21.58)	<0.001
% energy from CHO	36.04 (35.61–36.47)	35.68 (34.78–36.59)	0.49
% energy from proteins	20.56 (20.36–20.76)	20.85 (20.43–21.27)	0.23
% energy from lipids	40.51 (40.12–40.91)	40.88 (40.05–41.71)	0.44
Total CHO (g/dinner)	54.93 (54.08–55.77)	55.65 (53.88–57.42)	0.48
Simple CHO (g/dinner)	19.81 (19.37–20.25)	19.86 (18.92–20.79)	0.93
Total Proteins (g/dinner)	31.82 (31.38–32.26)	33.08 (32.15–34.01)	0.02
Animal protein (g/dinner)	22.58 (22.18–22.98)	23.61 (22.77–24.46)	0.03
Plant-based protein (g/dinner)	9.23 (9.06–9.39)	9.48 (9.13–9.82)	0.21
Lipids (g/dinner)	29.20 (28.67–29.73)	30.29 (29.18–31.41)	0.09
Monounsaturated fatty acids (g/dinner)	12.70 (12.45–12.95)	13.14 (12.62–13.66)	0.14
Polyunsaturated fatty acid (g/dinner)	5.48 (5.33–5.63)	5.85 (5.52–6.17)	0.05
Saturated fatty acid (g/dinner)	8.14 (7.96–8.33)	8.30 (7.92–8.69)	0.46
Fiber (g/dinner)	7.41 (7.25–7.57)	7.84 (7.51–8.17)	0.03
Ethanol MEN			
Non-consumer Ethanol	452 (72.7)	170 (27.3)	
Ethanol (g/dinner)	8.06 (7.42–8.70)	7.40 (6.28–8.52)	0.32
Ethanol WOMEN			
Non-consumer Ethanol	577 (74.5)	198 (25.6)	
Ethanol (g/dinner)	3.77 (3.35–4.19)	4.00 (2.97–5.02)	0.69

CHO = carbohydrates; g = grams. Adjusted means for center, sex, age at recruitment, educational level, smoking history, non-laboral physical activity (MET-h/week), BMI, % body fat, and hypertension.

**Table 3:** Association (OR, 95% CI) between sleep quality, chronotype, and chrono-nutrition and prevalence of Type 2 diabetes mellitus (T2DM) prevalence.

	OR (95% CI)	P-Trend
Time of sleep (h/free-days)	0.95 (0.84, 1.07)	
Time of sleep (h/work-days)	1.04 (0.91, 1.19)	
Sleep Quality		
Good	1 (Referent)	
Poor	2.90 (1.30, 6.28)	
Chronotype		
Early type	1.32 (0.96, 1.80)	
Normal type	1 (Referent)	
Late type	1.21(0.88, 1.64)	
Social jetlag (h)		
<0:30	1 (Referent)	0.41
0:30–1:30	0.87 (0.67, 1.14)	
>1:30	0.87 (0.52, 1.40)	
Number of eating occasions		
≤3	1.45 (0.91, 2.26)	0.11
4–5	1 (Referent)	
>5	1.16 (0.96, 1.41)	
Breakfast time (h)		
≤9:00	1 (Referent)	0.35
>9:00	1.15 (0.92, 1.44)	
Lunch time (h)		
≤14:00	1 (Referent)	0.39
>14:00–15:00	0.94 (0.75, 1.18)	
>15:00	1.36 (0.88, 2.06)	
Dinner time (h)		
≤21:00	1 (Referent)	0.31
21:00	0.97 (0.76, 1.24)	
>21:00	1.18 (0.91, 1.53)	

OR = Odds ratio; CI = Confidence Interval; h = hour. Logistic regression model adjusted for center, sex, age, educational level, smoking history, non-laboral physical activity (MET-h/week), body mass index, % body fat, and hypertension.

**Table 4:** Mutually adjusted logistic models between macronutrients by eating occasions and prevalence of Type 2 diabetes mellitus (T2DM).

Eating Occasion								
Breakfast			Lunch			Dinner		
Range	OR (95% CI)	P-Trend	Range	OR (95% CI)	P-trend	Range	OR (95% CI)	P-Trend
CHO (g/occasion)								
Q1 (<32.6)	1 (Referent)	<0.01	Q1 (<88.9)	1 (Referent)	0.40	Q1 (<36.8)	1 (Referent)	0.76
Q2 (32.6–43.9)	0.84 (0.62, 1.14)		Q2 (88.9–108)	0.93 (0.67, 1.29)		Q2 (36.8–48.1)	0.89 (0.65, 1.23)	
Q3 (44–54.9)	0.64 (0.46, 0.90)		Q3 (108.1–124)	0.76 (0.53, 1.08)		Q3 (48.2–57.7)	1.31 (0.96, 1.78)	
Q4 (55–69.3)	0.48 (0.33, 0.69)		Q4 (124.1–145)	0.83 (0.57, 1.22)		Q4 (57.8–72.2)	1.04 (0.76, 1.44)	
Q5 (>69.4)	0.40 (0.27, 0.59)		Q5 (>145.1)	0.89 (0.65, 1.22)		Q5 (>72.3)	1.10 (0.78, 1.54)	
Continuous (1-SD) *	0.75 (0.66, 0.85)		Continuous (1-SD) *	0.90 (0.80, 1.02)		Continuous (1-SD) *	1.02 (0.91, 1.13)	
Protein (g/occasion)								
Q1 (<8.2)	1 (Referent)	0.36	Q1 (<47.9)	1 (Referent)	0.29	Q1 (<21.7)	1 (Referent)	0.31
Q2 (8.2–11.2)	1.18 (0.85, 1.65)		Q2 (47.9–55.6)	0.94 (0.68, 1.30)		Q2 (21.7–28.1)	0.95 (0.69, 1.32)	
Q3 (11.3–13.7)	1.39 (0.97, 1.99)		Q3 (55.7–62.3)	1.12 (0.80, 1.58)		Q3 (28.2–34)	1.08 (0.77, 1.53)	
Q4 (13.8–17.2)	1.16 (0.79, 1.69)		Q4 (62.4–71.8)	1.07 (0.74, 1.55)		Q4 (34.1–41.8)	0.92 (0.63, 1.34)	
Q5 (>17.3)	0.89 (0.65, 1.22)		Q5 (>71.8)	1.35 (0.90, 2.05)		Q5 (>41.9)	1.26 (0.85, 1.89)	
Continuous (1-SD) *	1.05 (0.93, 1.19)		Continuous (1-SD) *	1.13 (0.98, 1.31)		Continuous (1-SD) *	1.08 (0.95, 1.23)	
Lipids (g/occasion)								
Q1 (<4.9)	1 (Referent)	0.11	Q1 (<34)	1 (Referent)	0.26	Q1 (<17)	1 (Referent)	0.30
Q2 (4.9–8.8)	1.15 (0.82, 1.61)		Q2 (34–42)	1.01 (0.74, 1.38)		Q2 (17–23.9)	1.17 (0.85, 1.62)	
Q3 (8.9–12.9)	1.62 (1.14, 2.29)		Q3 (42.1–49.7)	0.84 (0.60, 1.18)		Q3 (24–31.3)	1.35 (0.96, 1.89)	
Q4 (13–19.1)	1.29 (0.95, 1.75)		Q4 (49.8–60.3)	0.99 (0.69, 1.42)		Q4 (31.4–40.4)	1.39 (0.97, 2.00)	
Q5 (>19.2)	1.20 (0.87, 1.65)		Q5 (>60.4)	0.79 (0.52, 1.19)		Q5 (>40.5)	1.21 (0.80, 1.81)	
Continuous (1-SD) *	1.13 (1.01, 1.26)		Continuous (1-SD) *	0.90 (0.78, 1.04)		Continuous (1-SD) *	1.04 (0.90, 1.19)	

CHO = carbohydrates; Q = quartile. * One standard deviation increment of the control for T2DM was applied. (Breakfast: carbohydrates: 24.6 g, proteins: 6.4 g, and lipids 9.9 g; Lunch: carbohydrates: 33.4 g, proteins: 14.8 g, and lipids:16.1 g; Dinner: carbohydrates: 23.3 g, proteins: 12.8 g, and lipids 14.9 g). Mutually adjusted logistic regression model adjusted for center, sex, age, educational level, smoking history, non-laboral (PA, MET-h/week), body mass index class, body fat percentage, hypertension, sleep quality, alcohol intake and quantity, and total energy intake. Estimation of the OR was not modified by sex, smoking status, BMI, or sleep quality (Table S1).

## Data Availability

The data of this study is preserved by the EPIC-Spain research group. Data are subject to data sharing agreements and are not publicly available.

## Supplementary Materials

The following supporting information can be downloaded at https://www.mdpi.com/article/10.3390/nu16162598/s1, Table S1: Estimation of the modification of the OR by sex, smoking status, BMI, sleep quality, and chronotype.

## Abbreviations

CHNS, China Health and Nutrition Survey, CHO, carbohydrate; CI, confidence interval; DH-E, diet history questionnaire—ENRICA; EPIC, European Prospective Investigation into Cancer and Nutrition; h, hour; g, grams; MSFc, mid-sleep time corrected for sleeping on working and weekend days; OR, odds ratio; PA, physical activity; PSQI, Pittsburgh Sleep Quality Index; Q, quartile; T2DM, type 2 diabetes mellitus.
